# Supplementary material for: Visual Parameter Selection for Spatial Blind Source Separation
Source: Comput Graph Forum. 2022 Jul 29;41(3):157–68. doi: 10.1111/cgf.14530 (PMC9543588; doi:10.1111/cgf.14530)
Supplement: Supplementary file 1 — Supporting Information [file CGF-41-157-s002.pdf]

who	component	statement	score
VE1	insight	1	6
VE1	insight	2	7
VE1	insight	3	5
VE1	insight	4	6
VE1	insight	5	6
VE1	insight	6	7
VE1	insight	7	5
VE1	insight	8	6
VE1	time	9	6
VE1	time	10	6
VE1	time	11	7
VE1	time	12	5
VE1	time	13	7
VE1	essence	14	6
VE1	essence	15	6
VE1	essence	16	5
VE1	essence	17	6
VE1	confidence	18	6
VE1	confidence	19	6
VE1	confidence	20	NA
VE1	confidence	21	4
VE2	insight	1	7
VE2	insight	2	7
VE2	insight	3	6
VE2	insight	4	6
VE2	insight	5	7
VE2	insight	6	7
VE2	insight	7	7
VE2	insight	8	6
VE2	time	9	7
VE2	time	10	6
VE2	time	11	7
VE2	time	12	7
VE2	time	13	7
VE2	essence	14	6
VE2	essence	15	7
VE2	essence	16	7
VE2	essence	17	6
VE2	confidence	18	7
VE2	confidence	19	7
VE2	confidence	20	NA
VE2	confidence	21	NA
VE3	insight	1	6
VE3	insight	2	7
VE3	insight	3	7
VE3	insight	4	6
VE3	insight	5	7
VE3	insight	6	7
VE3	insight	7	7
VE3	insight	8	7
VE3	time	9	7
VE3	time	10	7
VE3	time	11	7
VE3	time	12	7
VE3	time	13	7
VE3	essence	14	6
VE3	essence	15	6
VE3	essence	16	5

	S	mean	std dev
insight	1	6.40	0.55
	2	7.00	0.00
	3	5.40	2.07
	4	6.40	0.55
	5	6.40	0.55
	6	6.80	0.45
	7	6.60	0.89
	8	5.80	0.84
time	9	6.80	0.45
	10	6.20	0.45
	11	6.80	0.45
	12	6.00	1.00
	13	6.80	0.45
essence	14	6.40	0.55
	15	6.40	0.55
	16	5.80	1.10
	17	5.60	1.14
confidence	18	6.60	0.55
	19	6.20	0.45
	20	**NA**	**NA**
	21	4.33	2.61

insight	*	6.35	0.98
time	*	6.52	0.65
essence	*	6.05	0.89
confidence	*	6.00	2.98

VE3	essence	17	5					
VE3	confidence	18	6					
VE3	confidence	19	6					
VE3	confidence	20	NA					
VE3	confidence	21	NA					
VE4	insight	1	6					
VE4	insight	2	7					
VE4	insight	3	2					
VE4	insight	4	7					
VE4	insight	5	6					
VE4	insight	6	6					
VE4	insight	7	7					
VE4	insight	8	5					
VE4	time	9	7					
VE4	time	10	6					
VE4	time	11	7					
VE4	time	12	6					
VE4	time	13	6					
VE4	essence	14	7					
VE4	essence	15	6					
VE4	essence	16	5					
VE4	essence	17	4					
VE4	confidence	18	7					
VE4	confidence	19	6					
VE4	confidence	20	NA					
VE4	confidence	21	3					
VE5	insight	1	7					
VE5	insight	2	7					
VE5	insight	3	7					
VE5	insight	4	7					
VE5	insight	5	6					
VE5	insight	6	7					
VE5	insight	7	7					
VE5	insight	8	5					
VE5	time	9	7					
VE5	time	10	6					
VE5	time	11	6					
VE5	time	12	5					
VE5	time	13	7					
VE5	essence	14	7					
VE5	essence	15	7					
VE5	essence	16	7					
VE5	essence	17	7					
VE5	confidence	18	7					
VE5	confidence	19	6					
VE5	confidence	20	7					
VE5	confidence	21	6					
